# Comparison of Shoulder Ultrasonographic Assessments between Polymyalgia Rheumatica and Frozen Shoulder in Patients with Bilateral Shoulder Pain: A Comparative Retrospective Study

**DOI:** 10.3390/jpm11050372

**Published:** 2021-05-03

**Authors:** Eun-Woo Park, Jang-Hyuk Cho, Chul-Hyun Cho, Duk-Hyun Sung, Du-Hwan Kim

**Affiliations:** 1 Dongsan Medical Center, Department of Rehabilitation Medicine, School of Medicine, Keimyung University, Daegu 42601, Korea; parkew1234@naver.com (E.-W.P.); jacob.chojh@gmail.com (J.-H.C.); 2 Dongsan Medical Center, Department of Orthopedic Surgery, School of Medicine, Keimyung University, Daegu 42601, Korea; oscho5362@dsmc.or.kr; 3 Samsung Medical Center, Department of Physical and Rehabilitation Medicine, Sungkyunkwan University School of Medicine, Seoul 06351, Korea; yays.sung@samsung.com; 4 Department of Physical Medicine and Rehabilitation, College of Medicine, Chung-Ang University, 102 Heukseok-ro, Dongjak-gu, Seoul 06973, Korea

**Keywords:** bursitis, painful shoulder, polymyalgia rheumatica, synovitis, tenosynovitis, ultrasound

## Abstract

This study aimed to assess and compare the ultrasonographic (US) pathologic findings in patients with polymyalgia rheumatica (PMR) and bilateral frozen shoulder (FS). We included 19 patients with clinically diagnosed PMR and 19 patients with stage II bilateral FS. The US evaluation included the assessment of subacromial-subdeltoid (SASD) bursitis, long head of biceps (LHB) tenosynovitis, and posterior and inferior glenohumeral (GH) synovitis. Unilateral SASD bursitis was noted significantly more frequently in PMR patients than in bilateral FS patients (*p* = 0.001). There were no significant differences in the incidence of unilateral LHB tenosynovitis and posterior GH synovitis between PMR and bilateral FS patients (*p* = 0.108 and *p* = 0.304, respectively). Unilateral inferior GH synovitis was more common among bilateral FS patients than among PMR patients (*p* < 0.001). Bilateral SASD bursitis and LHB tenosynovitis were noted significantly more frequently in PMR patients than in bilateral FS patients (*p* < 0.001 and 0.049, respectively). Significant differences were not observed in the incidence of bilateral posterior GH synovitis between PMR and bilateral FS patients (*p* = 0.426). Bilateral inferior GH synovitis was more common among bilateral FS patients than among PMR patients (*p* = 0.044). The US evidence for bilateral inferior GH synovitis without bilateral SASD showed high specificity (94.7%) with sensitivity (78.9%) for the diagnosis of bilateral FS. SASD bursitis, representing periarticular synovial inflammation, was more common among the patients with PMR than among the patients with bilateral FS. Inferior GH synovitis without SASD bursitis suggests FS rather than PMR in patients with bilateral shoulder pain.

## 1. Introduction

Frozen shoulder (FS) is a common pathologic condition of the shoulder joint capsule, which is characterized by progressive shoulder pain and the restriction of range of motion [1,2]. FS can develop bilaterally [1,2]. Approximately 20–30% of the patients affected develop the condition in the opposite shoulder [3,4]. Although the precise incidence of simultaneous bilateral symptoms in this condition is unknown, bilateral involvement is associated with a poor response to conservative treatments [5]. The diagnosis of FS is primarily based on medical history, physical examination, and negative plain radiography [2]. Often, it is difficult to differentiate idiopathic FS from other stiff shoulder conditions such as rotator cuff tear, calcific tendinitis, or inflammatory arthropathy [2,3,4]. In the case of bilateral shoulder involvement, it is important to differentiate between bilateral FS and polymyalgia rheumatica (PMR), as PMR is characterized by bilateral shoulder aching, stiffness, and is known to be associated with an inflammatory disease of the large blood vessels, i.e., giant cell arteritis [6,7,8]. An elevated level of inflammatory markers supports the diagnosis of PMR, whereas inflammatory markers have no role in the diagnosis of FS. [9] Although these two diseases are known to have distinctly different laboratory findings, 17.1% of patients were misdiagnosed with FS prior to a diagnosis of PMR owing to their similar clinical features [10]. Approximately 20% of patients with PMR have normal levels of inflammatory markers, whereas 30% of patients with idiopathic FS have elevated levels of high-sensitivity C-reactive protein (CRP) [11,12].

Musculoskeletal ultrasound has become increasingly useful in the diagnosis and treatment of rheumatic diseases as well as degenerative joint disease and peripheral nerve diseases [13,14,15,16]. According to the 2012 ACR/EULAR, the ultrasound (US) criteria for PMR include evidence of subdeltoid bursitis, biceps tenosynovitis, glenohumeral synovitis, hip joint synovitis, or trochanteric bursitis [9]. These findings can be non-specific and secondary to mechanical etiologies [14]. The role of imaging in diagnosing FS is still debatable. US can show effusion in the bicipital groove, thickened axillary pouch, and hypervascular hypoechoic tissue at the rotator interval in patients with FS [17,18,19,20,21,22]. Both PMR and FS can show similar findings in a shoulder US, suggesting tenosynovitis and glenohumeral synovitis [17,18,19,20,21,22,23,24,25]. However, the typical US findings have not been clearly established in either disease entity.

Considering the similarity of clinical findings and previously reported US findings for PMR and bilateral FS, the role of US in differentiating these two diseases should be clarified. This study aimed to compare the US findings of the shoulder joint in patients with PMR and bilateral FS.

## 2. Methods

### 2.1. Subjects

Patients with new onset of bilateral shoulder pain were referred from the shoulder pain clinic for the US evaluation at the musculoskeletal US clinic of the Department of Rehabilitation Medicine in a single tertiary hospital from March 2014 to February 2018. We retrospectively reviewed the medical records and US findings of 49 patients who were presumptively diagnosed with new onset PMR or bilateral FS by a single shoulder specialist (CHC, orthopedic surgeon) based on clinical features and blood tests. The patients with bilateral FS met the following criteria: (1) age ≥ 20 years, (2) bilateral shoulder pain with a limitation of passive movement to less than 30 degrees in two or more planes, (3) negative plain radiography, and (4) stage II FS as defined by Hannafin and Chiaia [26]. The patients with PMR met the following criteria: (1) fulfilled the 2012 ACR/EULAR classification criteria, and (2) underwent US examination before treatment with corticosteroids. We excluded the following conditions: rotator cuff pathology, previous peri-articular injection within 3 months, cervical myeloradiculopathy, myofascial pain syndrome, fibromyalgia, infection, chronic kidney disease, osteoarthritis, previous shoulder surgeries, high-energy trauma, and the presence of other combined inflammatory arthropathies such as rheumatoid arthritis (RA), gout, pseudogout, or spondyloarthritis.

Diagnostic US can exclude rotator cuff pathologies such as rotator cuff tendinopathy, calcific tendinitis, and a partial or full thickness rotator cuff tear. Of the 49 patients with a presumptive diagnosis of new onset PMR or bilateral FS, 11 patients with rotator cuff pathologies were excluded. Since a differential diagnosis between PMR and bilateral FS can be difficult at the time of the initial work-up, the final diagnosis was made retrospectively by a single shoulder specialist (CHC) at a 1 year follow-up based on the clinical course, change in inflammatory markers, and response to corticosteroids. Finally, 19 patients with new-onset PMR and 19 with bilateral FS were included in the analysis on the basis of their final diagnosis.

The patients’ demographic data, duration of disease, and their initial laboratory data were recorded, including erythrocyte sedimentation rate (ESR), CRP level, anti-nuclear antibody (ANA), rheumatoid factor (RF), and anti-cyclic citrullinated peptide (anti-CCP). This study received approval from the Institutional Review Board of Dongsan Medical Center (IRB No: 2020-10-028, approved on 16 October 2020).

### 2.2. US Protocol

US assessment was performed on both shoulders of each patient with PMR or bilateral FS. All US investigations were performed by a physiatrist sonographer (DHK) with an Accuvix V10 (Samsung Medison, Seoul, Korea) with a 5–13 MHz linear transducer. The operator was blinded to the clinical and laboratory findings.

The US shoulder evaluation was performed using a modification of the standard scanning method and particular attention was paid to detecting and assessing subacromial-subdeltoid (SASD) bursitis, long head of biceps (LHB) tenosynovitis, glenohumeral (GH) synovitis, and the presence of rotator cuff pathology [27]. With the patient arm in a neutral position, the bicipital groove was examined to detect any effusion in the LHB sheath. With the arm in the modified Crass position, the SASD bursa was examined. With the arm held in a neutral position, the posterior aspect of the GH joint, allowing visualization of the posterior labrum and the infraspinatus tendon, was scanned for evidence of GH effusion. In addition, axillary pouch thickness was measured to determine the presence of GH synovitis, with the patient supine, as recommended by previous researchers [18,28].

**Figure 1:**
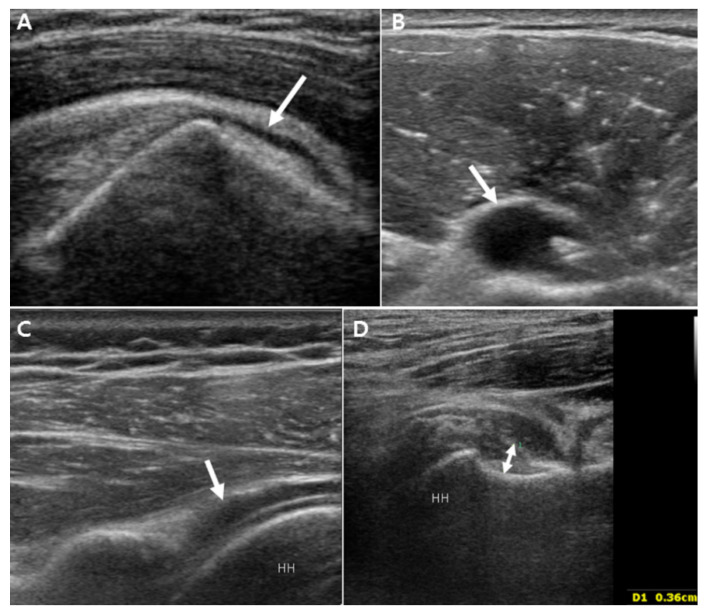
Illustrative grayscale ultrasonography images. (**A**) Subacromial/subdeltoid bursa (arrow) filled with fluid, (**B**) long head biceps tenosynovitis with effusion (arrow), (**C**) posterior glenohumeral synovitis with effusion (arrow), and (**D**) measurement of axillary pouch thickness to determine the presence of inferior glenohumeral synovitis (double arrow). HH, humeral head.

**Figure 2:**
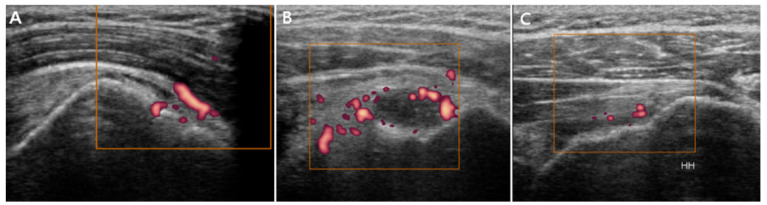
Illustrative power Doppler ultrasonography images. (**A**) Subacromial/subdeltoid bursitis, (**B**) long head biceps tenosynovitis, and (**C**) posterior glenohumeral synovitis. HH, humeral head.

SASD bursitis was diagnosed if the maximal thickness of the hypoechoic fluid-filled bursa was >2 mm [24]. LHB tenosynovitis was considered if the maximal thickness of the hypoechoic fluid around the biceps tendon was >2 mm [24]. GH synovitis was considered if the distance from the posterior labrum to the infraspinatus tendon exceeded 2 mm (posterior GH synovitis) or if the axillary pouch thickness was >3.5 mm (inferior GH synovitis) [24,29]. The axillary pouch thickness was measured as the distance from the bony cortex of the humerus to the outer margin of the capsule [18,28]. These four grayscale US inflammatory parameters were graded according to a dichotomous evaluation (absence or presence) (Figure 1). Power Doppler US was used to quantitatively evaluate the severity of inflammation at the LHB, SASD bursa, and posterior GH capsule (Figure 2). The power Doppler signal was subjectively graded on a semiquantitative scale (0 = absent or minimal flow, 1 = mild or single-vessel signal, 2 = moderate or confluent, and 3 = severe or vessel signals in >50% of the synovium area) [25]. Power Doppler was infeasible near the axillary pouch region owing to the pulsation artifacts caused by the axillary artery.

### 2.3. Statistical Analysis

SPSS 18.0 software for Windows^®^ (SPSS Inc, Chicago, IL, USA) was used for the analysis. Continuous variables are expressed as the mean and the standard deviation. Ordinal and dichotomous variables are expressed as frequencies and percentages. The Mann–Whitney U-test was used to compare mean values and Fisher’s exact test was used to compare frequencies. A value of *p* < 0.05 was considered to indicate statistical significance.

A power analysis indicated that a sample size of 16 patients in each group would be required to have a 90% chance of detecting, as significant at the 5% level, an increase in the SASD bursitis from 30% in the bilateral FS group to 80% in the PMR group.

## 3. Results

### 3.1. Demographic and Clinical Data

The clinical characteristics of the patients are shown in Table 1. The 19 patients with PMR included 8 men and 11 women with a median age of 67.7 ± 11.7 years. The mean duration of symptoms was 4.5 ± 1.4 months. One patient with PMR had a positive RF-antibody, but none had an anti-CCP antibody. Seven patients were positive for ANA and one patient had giant cell arteritis. One patient had normal ESR (normal reference: <25.0 mm/h) and two patients had an ESR below 40.0 mm/h. No patient had a normal CRP level (normal reference: <0.5 mg/dL). None of the patients with new onset PMR had another concomitant inflammatory arthritis.

The 19 patients with bilateral FS included 11 men and 8 women with a median age of 59.6 ± 8.6 years. The mean duration of symptoms was 5.4 ± 1.8 months. None were positive for RF or anti-CCP antibodies. One patient was positive for ANA. Ten patients had an abnormal ESR of up to 45.0 mm/h, and two patients had an ESR above 40.0 mm/h. Seven patients had an abnormal CRP level of up to 1.5 mg/dL.

The mean age of the PMR patients was greater than that of the bilateral FS patients (*p* = 0.025). Bilateral FS patients had diabetes more frequently than PMR patients (*p* = 0.003). The frequencies of abnormal ESR and CRP were significantly higher in PMR patients than in bilateral FS patients (*p* = 0.004 and *p* < 0.001, respectively).

### 3.2. Ultrasound Findings

All patients with PMR and bilateral FS received a bilateral shoulder US examination; *n* represents or the shoulder joints in the comparison of unilateral US pathologies between the two groups and the number of patients in the comparison of bilaterality of abnormal shoulder US findings.

#### 3.2.1. Unilateral Shoulder US Findings in PMR Patients and Bilateral FS Patients

Unilateral SASD bursitis, LHB tenosynovitis, posterior GH synovitis, and inferior GH synovitis were detected in 25/38 (67%), 29/38 (76%), 10/38 (26%), and 18/38 (47%) patients with PMR and in 11/38 (29%), 23/38 (61%), 11/38 (39%), and 34/38 (89%) patients with bilateral FS. Unilateral SASD bursitis was significantly more frequent in PMR patients than in bilateral FS patients (*p* = 0.001). There were no significant differences in unilateral LHB tenosynovitis or posterior GH synovitis between patients with PMR and bilateral FS (*p* = 0.108 and *p* = 0.304, respectively). Unilateral inferior GH synovitis was more common in bilateral FS patients than in PMR patients (*p* < 0.001). The thickness of the inferior GH synovium was significantly greater in bilateral FS patients than in PMR patients (*p* < 0.001; Table 2).

#### 3.2.2. Comparison of Bilaterality of Abnormal Shoulder US Findings in PMR Patients and Bilateral FS Patients

The incidence of bilateral SASD bursitis and bilateral LHB tenosynovitis was significantly higher among PMR patients than among bilateral FS patients (*p* < 0.001 and 0.049, respectively). Significant differences were not noted in the incidence of posterior GH synovitis in PMR patients and bilateral FS patients (*p* = 0.426). Bilateral inferior GH synovitis was more commonly noted among bilateral FS patients than among PMR patients (*p* = 0.044; Table 3).

#### 3.2.3. Power Doppler Findings

The distributions of the power Doppler US grades for SASD bursitis, LHB tenosynovitis, and posterior GH synovitis are shown in Table 4. The analysis revealed that the power Doppler grades of all three kinds of synovial pathologies in PMR patients were significantly greater than those in bilateral FS patients (*p* < 0.001, 0.007, and 0.015, respectively).

#### 3.2.4. Utility of US Evidence of Bilateral Inferior GH Synovitis without Bilateral SASD Bursitis for the Differential Diagnosis between PMR and Bilateral FS

The US evidence of bilateral inferior GH synovitis without bilateral SASD bursitis showed high specificity (94.7%) and sensitivity (78.9%) for the diagnosis of bilateral FS.

## 4. Discussion

In this study, we compared US findings between patients with PMR and patients with bilateral FS. SASD bursitis, representing periarticular synovial inflammation, was more common in patients with PMR than in patients with bilateral FS. LHB tenosynovitis and posterior GH synovitis, representing intra-articular effusion of the GH joint, were not significantly more frequent in PMR than in bilateral FS. Moreover, inferior GH synovitis, representing intra-articular synovitis, was more frequent in bilateral FS than in PMR. The analysis of the distribution of power Doppler grades revealed that those related to bursitis or synovitis in the PMR group were significantly higher than those in the bilateral FS group. These results suggest that although the optional components of an US may be nonspecific diagnostic criteria for PMR, SASD bursitis, representing periarticular synovial inflammation, is more common in patients with PMR than in patients with bilateral FS, and inferior GH synovitis without SASD bursitis suggests FS rather than PMR in patients with bilateral shoulder pain.

In 2012, the classification criteria for PMR, including optional use of US, were published [9]. Abnormal shoulder US findings included in the classification criteria were SASD bursitis, LHB tenosynovitis, and GH synovitis [9]. Several studies have tested the value of these criteria in cohorts of PMR patients, focusing in particular on the shoulder girdle; however, the results were inconsistent [24,25,29,30,31,32,33,34]. Cantini et al. suggested that US evidence of bilateral SASD bursitis can be used as a new diagnostic criterion for PMR based on high sensitivity (99.2%) and specificity (99.1%) [35]. Macchioni et al. reported that optional US use did not enhance the ability of the criteria to differentiate PMR from early-onset RA with PMR-like onset [33]. In previous studies, the control groups were heterogeneous, including healthy subjects and subjects with fibromyalgia, nonspecific shoulder conditions, and early-onset RA or other rheumatic diseases, in different proportions [24,25,29,30,31,32,33,34].

PMR typically affects the shoulder girdle bilaterally, with evidence of elevated acute-phase reactants, in patients aged >50 years [9]. The lack of a single reliable standard for PMR diagnosis may lead to some difficulty in distinguishing PMR from inflammatory arthritis, such as RA, or from spondyloarthritis or a non-inflammatory disorder, especially bilateral FS. Do et al. reported that 6 of 35 patients with a final diagnosis of PMR were previously diagnosed with FS [10]. FS commonly presents with nocturnal pain, suggesting an inflammatory cause, and may reveal elevated CRP levels [1,36]. A few reports have suggested that idiopathic FS may be associated with idiopathic frozen hip irrespective of rheumatic diseases, such as PMR or RA [37,38]. In contrast, up to 20% of patients with PMR may have an ESR < 40 mm/h [12,39]. FS is a common shoulder disorder with a lifetime prevalence of 2–5% among the general population. Considering the high prevalence of FS, it is more important in practice to distinguish between bilateral FS and PMR than to distinguish early-onset RA with PMR-like onset from PMR. To the best of our knowledge, previous studies have not assessed the differences in shoulder US findings between PMR and bilateral FS. We reported a high prevalence of shoulder US abnormalities, especially LHB tenosynovitis and inferior GH synovitis, in patients with FS. Considering the similarity of the clinical features of PMR and bilateral FS, this study suggests that bilateral FS patients can be overdiagnosed with PMR when using US optionally, in accordance with the 2012 classification criteria.

Although this study suggests a difference in US characteristics between PMR and bilateral FS, the most contrasting point between PMR and bilateral FS was the elevation in the level of inflammatory markers. No patient with PMR had normal serum levels of CRP, and only one patient with PMR had a normal ESR level. If a patient with bilateral shoulder pain has normal levels of inflammatory markers, it is reasonable for clinicians to consider FS instead of PMR, especially if they have inferior GH synovitis without SASD bursitis on an US.

Clinically, the differential diagnosis of bilateral shoulder pain between bilateral FS and PMR in patients with elevated inflammatory markers is important. In this study, a significant number of patients with FS showed abnormal levels of inflammatory markers, although the degree of the elevation of inflammatory markers was mild. Of 19 patients, 10 (52.7%) showed an increase in ESR and 7 (36.8%) patients showed an increase in serum CRP level. In clinical situations where inflammatory markers are elevated, our results will help to differentiate between these two diseases. Bilateral SASD bursitis on an US may suggest PMR rather than bilateral FS.

A previous study suggested that oral steroids seemed to be suboptimally effective in FS by providing a more rapid initial improvement in pain than that observed with no treatment, but with negligible differences by five months. The initial responsiveness to oral steroids might have a limited role in differentiating the two diseases [40]. A meticulous history of the presence of combined hip pain or morning stiffness and underlying diabetes, US characteristics of shoulder joints, and long-term monitoring of responsiveness to oral steroids may help to differentiate between the two disorders. In our study, 7 out of 19 patients with PMR were positive for ANA. Positive ANA in PMR is commonly observed [10,41]. The high prevalence of ANA positivity in PMR is probably because PMR mainly occurs in old age and involves high levels of inflammatory markers [42].

Since US has been used for PMR, attention has focused on the examination of the shoulder girdle. However, the US method of evaluating periarticular or intra-articular inflammation around the shoulder joints is not uniform. Jimenez-Paloop et al. considered the diagnosis of SASD bursitis or LHB tenosynovitis as established if the maximal thickness of the hypoechoic space was > 1.1 mm or 2.5 mm, respectively, and the diagnosis of GH synovitis as established if the distance between the humeral neck convexity and the joint capsule at the axillary pouch was > 3.5 mm [29]. In contrast, Ruta et al. diagnosed SASD bursitis or LHB tenosynovitis if the hypoechoic fluid within the SASD bursa or around the biceps tendon was > 2 mm and GH synovitis if the distance from the posterior labrum to the infraspinatus tendon was > 2 mm [24]. Recently, Suzuki et al. reported a semiquantitative scoring system for evaluating the severity of extrasynovial soft tissue inflammation of the shoulder using power Doppler in patients with PMR [25]. Further research is necessary to establish standardized US methods and criteria for optimal cutoff values. 

Optional US criteria for PMR include GH synovitis. Previous reports demonstrated that the prevalence of GH joint effusion tended to be lower than that of SASD bursitis and LHB tenosynovitis in patients with PMR [24,29,33,43]. Ruta et al. reported that GH effusion was detected in 7/60 (11.7%) shoulders, while SASD bursitis and LHB tenosynovitis were observed in 33/60 (55%) and 28/60 (46.6%) shoulders, respectively [24]. This study, consistent with previous reports, yielded results suggesting that the measurement of effusion around the LHB tendon is a more sensitive indicator of GH joint effusion (GH synovitis) than the measurement of posterior joint capsule effusion. This may be related to the depth of the target architecture and the difference in the dependent site of fluid collection.

This study has some limitations. The study design was retrospective and involved only a small number of patients. The estimated minimum sample size might be insufficient because the exact prevalence of SASD bursitis in the bilateral FS group is not well-known. The sex predominance of PMR is not typical, although this may be a result of the low numbers. We classified grayscale US inflammatory parameters using binary measurements (presence or absence). We did not evaluate the thickness of the coracohumeral ligament or the echotexture and vascularity within the rotator interval, which can be US parameters suggestive of FS [17,19]. We did not evaluate intra- and inter-observer reliability. We did not acquire a detailed history of medications taken before entry in this study. Administration of only a small dose of oral corticosteroids could have affected the US inflammatory findings [29,43]. Nevertheless, our study has some strengths. This study is the first to address the significant issue of the differential diagnosis of PMR with bilateral FS. Previous studies have focused on distinguishing PMR from RA [24,32,34]. However, considering FS is a common shoulder disorder and bilateral FS can be a common misdiagnosis before the diagnosis of PMR, it is meaningful that our study raised the issue of differential diagnosis of PMR and bilateral FS [10]. This study demonstrated some differences in US characteristics between PMR and bilateral FS. These US results might provide clues for the differential diagnosis of these disorders. Although optional US criteria were used for the classification criteria of PMR, our study highlights the necessity of standardized methods to evaluate SASD bursitis, LHB tenosynovitis, and GH synovitis.

## 5. Conclusions

Abnormal US findings of LHB tenosynovitis and GH synovitis are nonspecific findings that are commonly observed in PMR and in non-rheumatic diseases, such as FS. However, there are some points of discrimination between PMR and bilateral FS. SASD bursitis, representing periarticular synovial inflammation, was more commonly noted in patients with PMR than in patients with bilateral FS. Inferior GH synovitis, representing intra-articular synovitis, was more frequent in bilateral FS than in PMR. In patients with bilateral shoulder pain, the combination of elevated inflammatory markers and bilateral SASD bursitis on an US is suggestive of PMR, whereas inferior GH synovitis without SASD bursitis and with normal inflammatory markers suggests FS.

## Figures and Tables

**Table 1:** Demographic and clinical characteristics of the PMR and bilateral FS groups.

	PMR (*n* = 19)	Bilateral FS (*n* = 19)	*p*
Age, years	67.7 ± 11.7	59.6 ± 8.6	0.025
Sex, male:female	8:11	11:8	0.330
Disease duration, months	4.5 ± 1.4	5.4 ± 1.8	0.234
Diabetes, *n*	2	7	0.003
Abnormal ESR, *n*	18	10	0.004
Abnormal CRP, *n*	19	7	<0.001
ANA positive, *n*	7	1	0.017
RF positive, *n*	1	0	0.311
Anti-CCP antibody, *n*	0	0	1

PMR, polymyalgia rheumatica; FS, frozen shoulder; ESR, erythrocyte sedimentation rate; CRP, C-reactive protein; ANA, anti-nuclear antibody; RF, rheumatoid factor; anti-CCP, anti-cyclic citrullinated peptide.

**Table 2:** Unilateral synovial pathologies in patients with PMR and bilateral FS.

	PMR (*n* = 38)	Bilateral FS (*n* = 38)	*p*
SASD bursitis, *n* (%)	25 (67%)	11 (29%)	0.001
LHB tenosynovitis, *n* (%)	29 (76%)	23 (61%)	0.108
Posterior GH synovitis, *n* (%)	12 (32%)	9 (24%)	0.304
Inferior GH synovitis, *n* (%)	18 (47%)	34 (89%)	<0.001
Axillary pouch thickness, mm	3.3 ± 0.7	4.2 ± 0.7	<0.001

PMR, polymyalgia rheumatica; FS, frozen shoulder; SASD, subacromial-subdeltoid; LHB, long head of biceps; GH, glenohumeral.

**Table 3:** Bilateral synovial pathologies in patients with PMR and bilateral FS.

	PMR (*n* = 19)	Bilateral FS (*n* = 19)	*p*
SASD bursitis, *n* (%)	13 (68%)	1 (5%)	<0.001
LHB tenosynovitis, *n* (%)	14 (74%)	8 (42%)	0.049
Posterior GH synovitis, *n* (%)	5 (26%)	3 (16%)	0.426
Inferior GH synovitis, *n* (%)	9 (47%)	14 (74%)	0.044

**Table 4:** Distributions of power Doppler grades for three different sites around the shoulder joints in patients with PMR and bilateral FS.

	PMR (*n* = 38)				Bilateral FS (*n* = 38)				Fisher’s Exact Test
Grade	0	1	2	3	0	1	2	3	*p* value
LHB	4	14	13	7	12	5	19	2	0.007
SASD	7	11	18	2	24	8	6	0	<0.001
Posterior GH	22	14	2	0	33	5	0	0	0.015

PMR, polymyalgia rheumatica; FS, frozen shoulder; SASD, subacromial-subdeltoid; LHB, long head of biceps; GH, glenohumeral.

## Data Availability

The data presented in this study are available on request from the corresponding author.
